# Notices

**Published:** 1868-01

**Authors:** 


### NOTICES

THE NEW REPUBLIC, a Political Literary, Agricultural and Family newspaper is published weekly at Camden, New-Jersey, at two dollars a year in advance. H. L. Bonsall, Editor and Publisher; if the succeeding issues are as truthful, instructive and entertaining as No. 1, Vol. I, a prosperous future is in store for this new candidate for public patronage.

A new volume of THE LITTLE CORPORAL begins with the next number. The publisher has determined to continue his offer of the November and December numbers free to all new subscribers received during December. Terms $1.00 a year. Sample copy free if sent for before January 1. Address Alfred L. Sewell, Publisher of The Little Corporal, Chicago, Ill.

WEBSTER'S NATIONAL PICTORIAL DICTIONARY, 1040 pp., 8vo., published by G. & C. Merriam, Springfield, Mass. This volume has ben prepared upon the general principles of the large Dictionary, and with a full use of the materials of that work. It contains over SIX HUNDRED PICTORIAL ILLUSTRATIONS, under their appropriate words in the body of the work, with some thirty pages of grouped or classified cuts at the end;— the usual Tables, Classical, Scriptural, Geographical, Biographical, &c.; in addition to which, and as distinctive and valuable features of this Dictionary,

I. A GLOSSARY OF SCOTTISH WORDS AND PHRASES, very carefully prepared, and having received the revisory reading of PROF. WM. RUSSELL, the distinguished elocutionist, himself long a resident of Scotland, and peculiarly qualified to understand and express the true pronunciation of Scottish words. This Glossary will be especially valuable and acceptable to the many readers and admirers of Burns, Hogg, Sir Walter Scott, and other eminent Scottish writers.

II. A VOCABULARY OF PERFECT AND ALLOWABLE RHYMES, based upon the corresponding words in Walker's Rhyming Dictionary, but more copious and far more correct.

This work is not designed to supersede, or take the place of, Webster's New Unabridged Dictionary, in 1840 pages Royal

Quarto, containing 3000 Illustrations,—an indispensable work to every one who can afford it,—but to meet the wishes of many who, for convenience or economy, desire a more condensed and less expensive work. To such, whether students, families or business men, it is believed this volume will be found acceptable. Trade price, Six Dollars, in full sheep, marble edge, subject to the discount.

To Travellers. — The New Railway Time-Tables For Winter-Travel on all the Great lines of Railroads are published only in the American Railroad Guide.

Skating.—Several prominent physicians of New-York are inculcating the opinion that skating for girls and women is hurtful and dangerous; it is neither dangerous nor hurtful, but in every way healthful, exhilarating and delightful if young ladies would keep in mind the following important suggestions:

1. Do not pretend to go a skating, if the thermometer has fallen to twenty degrees Farenheit; nor if it has fallen to thirty and a cold, raw, damp wind prevails

2. Do not be on skates longer than an hour at a time

3. The moment you cease skating, go to the fire and rest; but while on the ice, keep in active motion.

4. When you start home, walk by all means, at least half a mile; thus, the circulation of the blood will be equalized and chilliness will be avoided.

5. Take an extra coat or cloak to be laid aside when you commence skating, and to be resumed the moment you cease.

All forms of exercise may be made hurtful by injudiciousness; if we take from our children all recreations which may be abused, we sign their death warrant. Exercise, activity, locomotion are to the young a necessity, and it is our wisdom to multiply all those pastimes which encourage bodily out-door activities, promote the circulation of the blood, the exhilaration of the animal spirits and the enlivenment of the mind The time of the young must be filled up; and if we fail to do this with innocent amusements and interesting recreations, they will either give themselves up to ennui, discontent and moodiness, or will fall into pastimes and practices which are in every way pernicious. Skating has all the enlivening influences of dancing, and none of its necessary immoralities; with the incalculable advantage of securing the breathing of a pure and invigorating out-door air, instead of the stifling heat and dust of the ball-room.

At McMillan's 5th Avenue 46th Street pond all possible facilities for warming and rest are given, and every family should have one or more representatives there, where the skaters will be select and disagreeable promiscuousness avoided.

Family Reading For 1868.—Parents owe it to themselves, their children, to the Church and to society to provide their households with some good religious newspaper, and if they can afford it, with some good secular paper, and a magazine of current literature, all costing less than ten dollars a year, and will avail more towards an elevating and refining influence in taste and mind and morals than many times that amount expended in dress and equipage and external accomplishments; let the daughter of a family show her force of character by learning to make her own dresses and trim her own bonnets, and with the saving supply herself and the family with a year's entertaining and instructive reading; and young man over fifteen! if you are worth a button you will do as much by throwing away your filthy tobacco and sham breast pin and brass watch guard and bogus finger jewelry; or if happily you are above these miserable vices of adornment and appetite, go work an hour a day for somebody, or do without one meal a day until the requisite amount is laid by to present your sisters and parents with a whole year's entertainment in the way of valuable periodical reading; it would be a good plan to send a dollar to one or more publishers and desire them to send their paper for as long a time as the money will pay for; during that time if the paper is a good one, you will not feel like parting with it; if not good, you can change for some other; all that you have to do is to write the name of the paper and the town in which it is published on the envelope, put on a stamp, enclose the dollar, with a note thus, "please send your paper for as long as the enclosed dollar will pay for to the address of John Smith, or whatever else may be your name, then write distinctly the name of the post office where you get your letters, the name of the county and state, seal with a wafer and deposit it in the post office yourself, saying nothing to any one, and in nine hundred and ninety-nine times out of a thousand it will not fail to reach its proper destination, such as been our experience; in twenty years we have never lost a dollar by sending it through the mail thus from the New-York post office. The publications which we now recommend are among our exchanges and from the four largest cities in the United States. because the papers from these points are of more general interest to our widely scattered readers, "The Examiner," New-York; "The Watchman and Reflector," Boston, Mass., are long established Baptist papers. The Evangelist of New-York city, The Presbyterian of Philadelphia, The Banner of Pittsburgh, Pa., The North Western Presbyterian of Chicago, Illinois, are Presbyterian papers; The Christian Advocate and the Methodist of New-York; Zion's Herald, Boston, and The Methodist Home Journal of Philadelphia are Methodist papers. The New-York Observer may be advantagously taken in any Christian family, or in any cultivated family whether religious or infidel; it is ably conducted and is of sterling worth; it is rich and too stingy to exchange with us; but as it has done the Church and the country good service for many years, we heartily commend it to the trial of any of our readers who may not have taken it hitherto.

Among the secular weeklies which are always filled with a large amount of valuable reading are The New-York Weekly Post; The Weekly Bee of Sacramento City, California is always filled with safe and valuable reading matter; so is The Journal of Commerce of Cincinnati, Ohio; The Weekly Commercial of Baltimore, and The Chronicle of Augusta. Georgia.

Harper's New Monthly Magazine, $4 a year, is known every where and with one exception, we heartily commend it to an intelligent public; its first numbers created a prejudice in our minds, which has never been removed, and the fault has been repeated occasionally since; it reminded us of the Methodist circuit riders we sometimes heard in our youth, "ayont the Alleghanies"; it seemed utterly impossible for any one of them ever to preach a sermon on any subject in which, in some way or other, he did not manage to lug in John Calvin for a kick; as if his mission were to annihilate the Divine, instead of the Devil; perhaps the first editor of Harper was a veteran circuitrider, elevated a mite, for he seemed determined to demolish Presbytery, and now and then he takes another turn, like Sancho against the windmill, but the mill nevertheless stands. We vote that every man, woman and child be permitted, in the matter of religion, "to sit under his own vine and fig-tree", and worship God according to the dictates of his own conscience; the same space devoted to the annihilation of Calvinism in Harper's New Monthly Magazine would have been employed more acceptably to the mass of its readers in the advocacy of the great principles of the Christian religion, which are common to all the sects of the Church.

As a means of inducing families to procure for themselves valuable reading for 1868, we offer the Journal at a low rate in combination with other publications.

The Atlantic Monthly; Harper's New Monthly Magazine; Harper's Illustrated Weekly; Harper's Weekly .Bazar of Fashion; Putnam's New Monthly; are each four dollars a year; "The Galaxy", New-York, $3. 50; Godey's Ladies' Book, $3. The Philadelphia Presbyterian, O. S., $3; for the price alone of either of these publications, sent to "Hall's Journal of Health", No. 2 W. 43d Street, New-York, in the form of a post office order, or bank check, such publication will be sent for one year with this Journal; thus throwing in the price of the Journal for nothing; when not otherwise ordered, the Journal will be sent from January 1868 and the other publications from the date of the letter; or beginning of the current volume.

PUBLISHER'S UNIFORM TRADE LIST DIRECTORY, by Howard Challen, 1308 Chestnut Street, Philadelphia.

This work, devoted to the special interests of publishers, is now being revised. Will contain the complete list of books published by over 200 leading Houses. It is designed to save publishers the expense of printing, and sending booksellers their separate lists, and to enable booksellers, librarians, and book buyers to refer readily to any list desired to make up orders by. No work similar to it or as comprehensive has ever been published in this or any country. It is as indispensable to the publisher to contribute his list to its columns to be recognised as being in business or having works of merit, as it is to the bookseller and book buyer to posses it, to find in its pages any book wanted by him. In addition to this it contains a vast amount of information respecting publishers, newspapers, stationery, &c., which will be of enterest to every literary man. No library, public or private, or any person in the book, stationery, or printing trades, can afford to do without it, as the time and money saved by its information will amply compensate for the price charged.

Mr. Howard Challen of Phila. the compiler, has in press an Alphabetical Catalogue, of all books published from January 1866 to January 1868, and desires any publisher or author who has issued any book during that period, to send full title, size, price, &c., &c.

---

NEW SUNDAY SCHOOL PAPER.—THE CHILDREN OF THE WEST. —Seven reasons, among others, why this paper should be put into the hands of every child that can read:

1. It is one of the very best Children's Papers published.
2. It offers a greater variety of attraction than any other.
3. It is the handsomest, and offers greater inducements to subscribers.
4. It is not only attractive to children, but imparts instruction of the most useful kind.
5. It is calculated to lead the young to Christ.
6. It will prove a great auxiliary in establishing and sustaining Sabbath Schools.
7. It is as cheap as can be asked. Only 50 cents a year.

☞ Send for specimen. Address—Wells & Courtney, Mount Lebanon, La.

Every Farmer who makes any pretentions to progress will take some agricultural publication; among these, the cheapest, best and most reliable monthly issue is The American Agriculturist of New-York city. The Country Gentleman of Albany, New-York, is a veteran weekly; and is edited with great care, discrimination and ability; The Gardener's Monthly of Philadelphia, Pa., is adapted to every family who has a garden, an orchard or a farm. We rather think that the best weekly secular newspaper in the United States suited and suitable for every household is The Germantown Telegraph, Pa., near Philadelphia; it is one of the papers which we never allow to pass from our hands without opening. Among the more stately periodicals or rather magazines, proper, are the Atlantic Monthly, $4 a year, Boston, Mass.; the very ablest pens in the nation are employed in making up its pages, and yet we do not commend it except to strong minded men and women; it is not a safe publication for growing up children; that is, it is not always safe, because every now and then there comes out an article unfriendly to the Christian religion,. this has always been its fault from the very first number; most of these articles are from the pens of Gail Hamilton, Harriet Beecher Stowe and Dr. Holmes, that is to say Dr. of Bones, but not of Physic; we do not think he could cure a goose of the tooth ache to save his life; in practical medicine he is a mountebank, in our opinion; we don't think he practices medicine at all, and for the best reason, he don't know how, hence he has made himself simply ridiculous from time to time, in decrying the efficacy of medicine in disease; and his theology is worse than his physic; a man who gives poison now and then in physic and in sentiment, is more dangerous than he who always gives poison. Can't Stowe and Holmes, and "Hamilton", with all their splendid powers and talents find something better to do than holding up Puritanism and the clergy and religious sects to ridicule; the Atlantic Monthly does itself very great injustice by the admission of these occasional "hits and digs" which are an insult to the understandings and consciences of nine-tenths of its readers.

The American Tract Society, 150 Nassau Street, New-York, has published three interesting little books for children THE DESERTED HEROINE, or the Wanderer Brought Home, 32 pp. DEAN PROCTOR, The Fine Scholar and His Rivals, 72 pp., and NELLY AND HER SISTER, or The Two Paths, by the Author of Phil Kennedy, 192 pp. The same Society has published a multitude of other books, at very cheap rates, well calculated to interest and instruct families gathered around the fire-side of winter evenings.

PUTNAM'S MONTHLY is received and meets with a cordial welcome from the Press; and if the same discernment presides over the selection of the contributions, as prevailed when the first number was issued fourteen years ago, the revived publication will take a deep and wide hold on a cultivated and discriminating public. We ask Mr. Putnam one question: Will you recommend yourself to a generous, liberal and appreciative constituency, by issuing a Magazine which will respect the religious sentiment of the country, of all classes and sects; which will not insult your Christian readers as the Atlantic Monthly has repeatedly done; and which will not be giving Presbyterians an intended annihilator, every now and then, as Harper's New Monthly Magazine has done? We think that any periodical, which, by being ably conducted in the main, will use its influence now and then against the religious sentiment of its readers, does not deserve public respect; for it is simply doing a very mean, underhanded thing; and we shall expect that the Atlantic and Harper will hereafter have more sense, and abate incontinently the moral nuisance which they have been committing! The Harpers are good Methodists, and Ticnor and Fields are very clever fellows of the big, broad, Universal Church, but if these men at any time find themselves in a belligerent mood, let them turn the muzzles of their artillery in the direction of down yonder.

But we always get off the track; we were speaking of the able men whom Putnam chose for his first number, and it is sad to think, how, in the space of fourteen years, so many have been called to that Bar where they must give an account of their stewardship for the talents which were given them for use in elevating mankind. William North, Fitz James D. O'Brien, Rev. Dr. Hawkes, Thoreau, Dr. Bethune, Dr. Baird, Rev. J. H. Hanson, William S. Thayer, Henry W. Herbert, Richard Hildreth, Mariah Lowell and others, all gone! and at the end of another fort-night of years, how many pens will have slept the sleep which knows no waking, which now every month electrify the public mind with all that is new and brilliant, and sparkling, and grand; may every one of all that shall be called from the stage of action, be ready to give in his "account with joy and not with grief."

Now we are going to give a "notice" of our own Journal, which we pronounce the most useful publication of the kind in the world; as proof, there is not another like it on the habitable globe; of course then it is the best; it is always on the best side of all questions, for example the Bible, the clergy and of woman-kind; it seeks to prolong the lives, happiness and health of every body, by showing how to avoid sickness and attain a cherry, beautiful old age, free from all bodily pain and suffering, and distress. As the result of the practice of our principles we don't remember to have had but a single ache in thirty years, nor to have lost a meal for want of an appetite, nor to have closed our office for a single hour on account of sickness. We know that such a result has come from the goodness of the Infinite One, arising however from the carrying out of Cromwell's principles, "pray to the Lord and keep your powder dry," if you want to gain the battle on a rainy day, and he did. Here we are in our office from morning until night, and from night until morning, from one year's end to another, engaged in "reading, writing and arithmetic," prescribing, and listening to the long rigmaroles of stories of the unfortunate sick; scarcely going outside of the city limits once a year; visiting nobody; not having a moment to spare from day-light until nine o'clock, when we jump into bed; catch us trotting to a five-mile-post and then back again before breakfast; or cutting monkey capers in a gymnasium two hours a day; or playing fisticuffs at imaginary wind-mills; or splashing around in cold water; we would'nt wash our face and hands in cold water, even of a mid-summer's morning for a premium ; we should feel dirty all day after-wards; cold water don't make the hands clean; the only legitimate use of cold water which we can think of at present is to drink when one is thirsty; turn mill-wheels and float ships; and then, this way of scrubbing the skin by the hour with crash-towels, coarse brushes and all that nonsense, as if it were desirable to make man's hide as tough and horny as that of a rhinoceros; what's the use of it? it's against nature and all common sense. Reader, what's the use of eating like a pig, and then have to work like a nigger—that is a freed-man, to get rid of the surplus; wouldn't it be better to eat less, and have no surplus to work off? eat only as much as nature requires, then take your ease, that's just like what a pig does; you don't find him trotting round his pen by the hour to work off a surfeit; a pig don't surfeit himself; he has too much sense for that; he simply eats what he wants, then stops and lies down and takes it easy; but what does man do, with his magnificent mind, so towering above the humble instinct of the poor brute? he eats more than he wants, as you, reader, have done many a time ; you have many a time eaten when you did not want any thing; many a time have you coaxed your appetite with brandy and whiskey, and red-pepper, as hot as fire, and pickles, and filthy "bitters" and made a confounded ass of yourself just for the good of a thing while it was passing down your gluttonous throat; pshaw! you ought to be sick ; no wonder you go around among your friends whining and mewing like a sick kitten, enumerating your aches and pains, and ailments, and wasting other people's time in listening to your pitiful lucubrations; to take you at your word, one would suppose you were at the death's door, and couldn't possibly live a week; and yet you have been at the same old thing for years ; get out!

It's all Tom-foolery about people killing themselves with hard study, by professional labor; if a man gets as much sleep as nature needs in every twenty-four hours, he will be as lively as a cricket at four-score, if he will only eat, and eat wisely. The papers have it now, that Spurgeon is about being laid on the shelf from excess of labor; don't believe a word of it; that's only a kink that the supple London Doctors have put into his head; if he had consulted us, as he ought to have done, we would simply have said, "go it, Spurgy," work while the day lasts and the fields are white, and while your hand is in; if you go to sleep, and rest on your oars, as young preachers do, when they marry rich wives, you will, when you wake up, find that it was a Delilah's blandishment and that the locks of your strength are gone. Only think of Paul being advised to rest; to do nothing; he would have thundered out "as much as in me lies, I will preach the Gospel, if I hang for it." Do you think the impulsive Peter could have been persuaded to take a sea voyage for the sake of his health, when souls were perishing? Would the tender and loving John have been willing to have laid aside his work to take a course of shower-baths, or movement-cures, of base-ball or nine-pins? or that grand old worker, John Wesley; why, if you had said to him he must rest a while, with all his meekness he would have told you, "go to grass, I'd rather wear out than rust out." No, no; the way for a minister to recuperate, is to work on; stop him suddenly, and like the puffing locomotive he will be exploded into smithereens, that is giblets, that is, into splinters, into infinitissimal atoms; if Spurgeon don't take this Journal, we hope some subscriber in London or Port Natal or Burmah will send him a copy, and let him have a piece of our mind; that it is not the variety of his occupations which has impaired his health; it is oneness of employment which oftener crazes the brain or prostrates its powers; the more different things a man attends to, the less likely is he to receive injury; and to stop any machine in its busy whirl instantly, is to destroy it. Mr. Spurgeon has become ill by a faulty method of eating, sleeping and exercise, which he could not have fallen into, if he had been a constant reader and doer of the words of this Journal. But since he is sick, he must not be neglected; let him turn Methodist circuit-rider in England and preach once every day and Sunday too, under the name of John Smith, so that the housewives on his way may not feed him to death; let him send out his appointments one day ahead, eating at three regular times a day, wherever he may happen to be at the time; but making the last meal of the day on a piece of bread and butter, and a cup of hot tea, and in less than six months, Richard will be himself again. But we began this with the intention of saying that every household in this nation would be benefitted by taking Hall's Journal of Health for 1868. But it is human nature to do what will injure itself, willingly, promptly, universally; but when it is wanted to pursue a course which will elevate and bless, it must be hired, so we hereby offer the public as follows:

We will send Hall's Journal of Health for 1868 without charge, to any one who will send by post office order, check or draft the price of either one of the following publications, either to "The Publisher of Hall's Journal of Health, No. 2 West 43 Street, New-York", or to the respective publishers, in either case stating that it is in accordance with this offer:

The Atlantic Monthly, Harper's New Monthly Magazine, Harper's Illustrated Weekly, Harper's Weekly Bazar of Fashion, Putnam's Monthly, all of which are four dollars a year; and Godey's Lady's Book; Moore's Rural New-Yorker; The Philadelphia Presbyterian; The Scientific American, the most valuable secular weekly in the nation for families, manufacturers, mechanics and gentlemen; these are three dollar publications; by accepting this offer, the reader can have either of the above publications at their regular prices, and Hall's Journal of Health thrown in, free, gratis, for nothing, for one whole year! But after January 1st 50 cents additional.

### To All Farmers.

Moore's Rural New-Yorker is three dollars a year; this will be sent with Hall's Journal of Health for 1868 for 3½ dollars; whatever farmer, who has two ideas to spare, does not take up with this offer, will shut his eyes to his very best physical and pecuniary interests; two such publications will afford him better and cheaper reading than can be obtained for the same sum anywhere else on the habitable globe. We do not expect this offer to be accepted by ten thousand farmers, because we do not think that there are that many who have much sense; as a class, they have but two ideas and one habit; the ideas are prices, weather; habit to work their wives to death; fact, solid serious fact; but we want to change that by making them the above offer, for they are "death" on cheap things; only let them know that a thing is offered below cost, and they will buy it, whether they want it or not, if they have the money to spare; but nine out of ten are heels over head in debt and will be, till their dying day; the more intelligent of them, men of force, men of culture and progress, believe in the value of agricultural papers: believe in the value of labor-saving machines, of mowers and reapers, and corn planters; these are the men who purchase no more land than they can pay for and cultivate to the best advantage; and they are the men who are ready to purchase an adjoining "piece," when it is offered at a bargain; these are the men who always have money to pay for books and magazines and agricultural weeklies, and who consider their wives the best hands on the place, and favor them, and look up to them, and love them, and lighten all their loads with a sympathy a deference, a respect and an affection which is delightful to witness, and which will never fail to be rewarded by domestic happiness, by pecuniary success and social elevation; all honor to the farmer who considers his wife the Pearl of the place, the light of his eye and the jewel of his heart; who sees in her for all after-life the sweet and loving and trusting bride of the wedding-day.

